# Histone H2B Mutations in Cancer

**DOI:** 10.3390/biomedicines9060694

**Published:** 2021-06-19

**Authors:** Yi Ching Esther Wan, Kui Ming Chan

**Affiliations:** 1 Department of Biomedical Sciences, City University of Hong Kong, Hong Kong, China; estherwan2-c@my.cityu.edu.hk; 2 Key Laboratory of Biochip Technology, Biotech and Health Centre, Shenzhen Research Institute of City University of Hong Kong, Shenzhen 518172, China

**Keywords:** oncohistone, histone mutation, epigenetics, cancer epigenetics, H2B

## Abstract

Oncohistones have emerged as a new area in cancer epigenetics research. Recent efforts to catalogue histone mutations in cancer patients have revealed thousands of histone mutations across different types of cancer. In contrast to previously identified oncohistones (H3K27M, H3G34V/R, and H3K36M), where the mutations occur on the tail domain and affect histone post-translational modifications, the majority of the newly identified mutations are located within the histone fold domain and affect gene expression via distinct mechanisms. The recent characterization of the selected H2B has revealed previously unappreciated roles of oncohistones in nucleosome stability, chromatin accessibility, and chromatin remodeling. This review summarizes recent advances in the study of H2B oncohistones and other emerging oncohistones occurring on other types of histones, particularly those occurring on the histone fold domain.

## 1. Introduction

**Figure 1:**
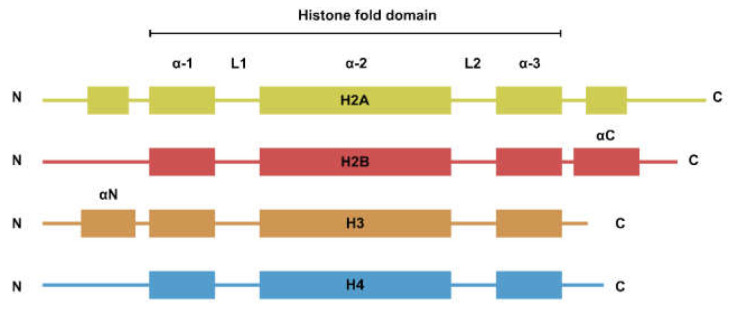
Secondary structure of the four core histones. Histones generally consist of two disordered tails bracketing the histone fold domain and some additional structured fold unique to each type of histone. Helices are represented by rectangles and loops are represented by lines.

The nucleosome is the basic repeating unit of the chromatin. Each nucleosome consists of two copies each of H2A, H2B, H3, and H4. While the four types of histones have dissimilar amino acid sequences, their secondary structures can be generalized to a histone fold domain flanked by two disordered tail domains (Figure 1). The tail domains are rich in lysine residues that are subjected to post-translational modifications such as methylation, acetylation, and ubiquitination. Histone modifications are involved in the regulation of a plethora of biological processes such as transcription and DNA damage repair [1].

**Figure 2:**
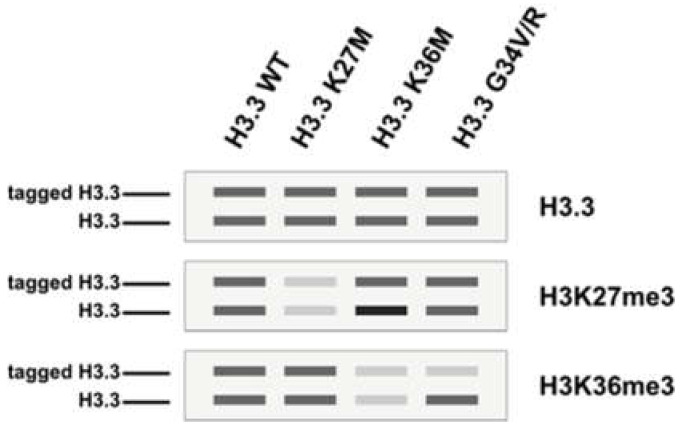
Schematics of immunoblots depicting the trans-inhibitory effect of H3K27M/H3K36M and cis-inhibitory effect of H3G34V/R. A tagged version of H3.3 (wildtype or mutated) was expressed in a human cell line followed by immunoprecipitation of the tagged H3.3 to retrieve mono-nucleosomes containing both endogenous H3.3 and tagged H3.3 (heterotypic nucleosome). For H3K27M and H3K36M (lane 2–3), the reduction of H3K27me3/H3K36me3 are observed on both copies of H3.3 (trans-inhibition). In contrast, the reduction of H3K36me3 is observed only on the tagged H3.3 (cis-inhibition). Of note, the expression of H3K36M also leads to an increase in H3K27me3 on endogenous H3.3. This figure is adapted from [10,15].

Oncohistone mutations are defined as clustered mono-allelic missense mutations that often affect only one of the histone genes (human histones are polygenic in nature; all four histones are encoded by at least fifteen genes), the expression of which exhibits oncogenic features [2,3]. Oncohistones have been an active area of research since 2010, starting with the identification of H3K27M (Histone H3 Lys27-to-Met missense mutation) and H3G34V/R in diffuse intrinsic pontine gliomas [4,5,6], closely followed by the report of H3K36M in chondroblastomas [7] and head and neck squamous cell carcinomas [8,9]. Although these mutations are found in diverse cancer types, they converge functionally to perturb histone post-translational modification and lead to aberrant gene expression. H3K27M is a dominant negative inhibitor of EZH2, the lysine methyltransferase for H3K27 methylations. The expression of the H3K27-to-M mutant results in trans inhibition of H3K27me3 and the concomitant loss of transcriptional silencing [10,11,12,13]. Acting in a similar manner, H3K36M inhibits SETD2, and the expression of H3K36M mutant histones leads to reduction of H3K36me2/3 in trans [14,15]. H3G34 mutations are different from H3K27M and H3K36M in that they inhibit H3K36me3 in cis [16] (Figure 2). For a detailed review of H3 mutations in cancer, please refer to references [2,3,17,18,19].

Recent efforts to catalogue histone mutations in cancer have vastly expanded the list of potential oncohistones with mechanisms beyond disruption to histone modifications. Utilizing patient data on cBioPortal [20] and MSK-IMPACT (Memorial Sloan Kettering-integrated mutation profiling of actionable cancer targets) [21,22], Nacev et al. [23] and Bennett et al. [24] reported more than 4000 missense mutations occurring in core histone genes, revealing functional convergence of many of the mutations. Some of the most prevalent mutations occur in the globular histone fold domain and are situated in regions important for the structural integrity of the nucleosome, leading to speculation that oncohistones might impede cellular processes beyond histone modifications. This review summarizes recent studies on histone fold mutants occurring in H2B including H2BG53D, H2BE76K/Q, and H2BE113K mutations.

## 2. H2BG53D Mutation

H2BG53D was identified in 0.07% of all of the cancer patients on cBioPortal. Cancers with H2BG53D mutation include, but are not limited to, pancreatic cancer, glioblastoma, prostate cancer, and lung cancer [23,24]. Long before the identification of H2BG53D in cancer patients, a homologous mutation in fission yeast (htb1-G52D) was reported to cause disruption to gene silencing in the heterochromatic region and defective chromosome segregation [25].

### 2.1. H2BG53 Is Located at the Histone/DNA Contact Point on the Nucleosome

**Figure 3:**
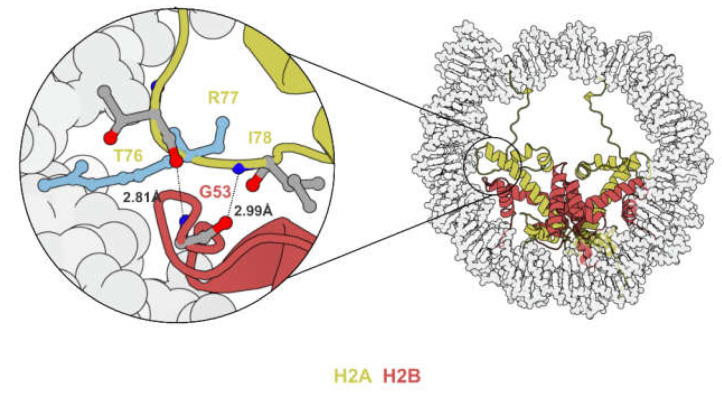
Illustration showing the H2A–H2B L1–L2 DNA binding region. H2AR77 (light blue) inserts into the minor groove of DNA. H2AT76 and I78, the two residues flanking H2AR77, form hydrogen bonds with H2BG53 (PDB code 2CV5).

DNA wrapping around the histone octamer is held in place by arginine residues projecting into the minor grooves of nucleosomal DNA [26]. Electrostatic interactions formed around these histone/DNA contact points stabilize the nucleosome and are barriers to processes that require unwinding of DNA. Since glycine-53 of histone H2B (H2BG53) is located in close proximity to one such histone/DNA contact point (H2AR77) [27,28,29] (Figure 3), the substitution of the neutrally charged glycine to negatively charged aspartate (H2BG53-to-D) was hypothesized to weaken the electrostatic interaction and thus facilitate processes such as nucleosome sliding and transcription. Of note, H2AR77 mutations were also identified in 0.03% of patients on cBioPortal [24].

### 2.2. H2BG53D Nucleosomes Facilitate Nucleosome Sliding and Are More Susceptible to Transcription

**Figure 4:**
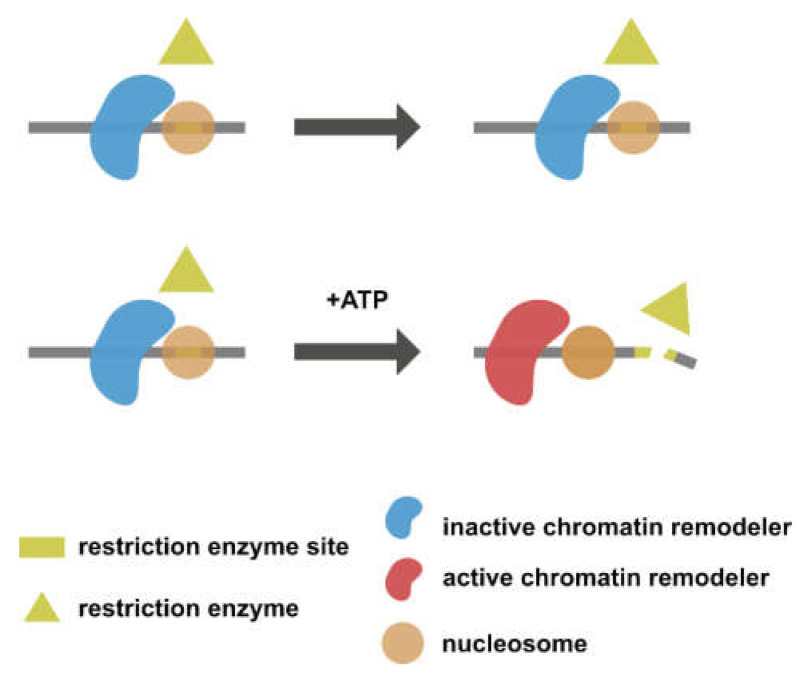
Restriction enzyme accessibility assay evaluating chromatin remodeling activity. The chromatin remodeler remains inactive in the absence of ATP. The restriction enzyme cut site is hence protected by the octamer (upper panel). The addition of ATP activates the chromatin remodeler, resulting in nucleosome sliding and exposure of the cut site (lower panel).

Bagert et al. [30] conducted a restriction enzyme accessibility assay to investigate how H2BG53D affects nucleosome sliding mediated by an ATP-dependent chromatin remodeler. In a restriction enzyme accessibility assay, nucleosomes are reconstituted with DNA containing a restriction enzyme recognition site. The restriction enzyme site remains inaccessible in the absence of an active chromatin remodeler (Figure 4, upper panel). In contrast, when the chromatin remodeler is activated by the addition of ATP, the restriction enzyme site will be exposed and cut by restriction enzymes as a result of nucleosome sliding (Figure 4, lower panel). The restriction enzyme accessibility assay conducted with an H2BG53D-containing nucleosome revealed an elevated remodeling rate, suggesting that H2BG53D might facilitate transcription in cooperation with chromatin remodeling machineries.

The in vitro transcription elongation assay performed by Wan et al. [31] showed that RNA polymerase II progresses quicker on a DNA template containing a H2BG53D nucleosome compared to one that contains a wild-type nucleosome. A single-molecule optical tweezer assay suggests that the increased efficiency by which RNA polymerase II passes through H2BG53D nucleosome is a result of weakened interaction between nucleosomal DNA and the histone octamer.

While H2BG53D’s effect on nucleosome sliding has yet to be examined in a cell-based system, H2BG53D-assocatied transcriptional alterations and its contribution to oncogenesis have been characterized in pancreatic cancer cell lines [31,32].

### 2.3. H2BG53D Alters Transcription in Pancreatic Cancer Cell Lines

Wan et al. [31] reported the presence of the H2BG53D mutation in pancreatic ductal adenocarcinoma (PDAC, 6.8%), lung squamous cell carcinoma (1.1%), and glioblastoma multiforme (1%). To study the biological consequence of H2BG53D expression at a physiological level, genome editing mediated by CRISPR/Cas9 was employed to construct stable cell lines that express FLAG-H2B/FLAG-H2BG53D from the endogenous *HIST1H2BO* locus. Contrary to the study in yeast where H2BG53D expression inhibits growth [30], H2BG53D expression from its endogenous promoter does not lead to a change in the proliferation of PDAC cell lines. H2BG53D expression in PDAC cell is, however, associated with a gain of migratory properties, indicating changes in gene expression in H2BG53D cell lines. Indeed, transcriptome profiling revealed that the gene expression profile of FLAG-H2BG53D cell lines is distinct from that of isogenic FLAG-H2B cell lines. Differentially expressed genes in H2BG53D cell lines are overrepresented in pathways such as the “Rap1-signaling pathway”, “proteoglycans in cancer”, and “ECM-receptor interaction”, all of which are relevant to oncogenesis [32].

Although RNA-seq provided strong evidence for gene expression changes in H2BG53D cell lines, it does not distinguish between transcriptional and post-transcriptional alterations. To investigate whether H2BG53D does indeed alter gene expression on the transcriptional level, Wan and colleagues [32] profiled the nascent transcript level in H2BG53D cell lines by Precision Run-On sequencing (PRO-seq) [33]. Consistent with in vitro transcription assay showing increased pol II passaging in H2BG53D nucleosomes, differentially transcribed genes in PRO-seq were dominated by upregulated genes.

### 2.4. H2BG53D Target Genes Are Upregulated and Are Implicated in Oncogenesis

The results of RNA-seq and PRO-seq from Wan et al. [32] suggest that H2BG53D expression is associated with elevated transcription. To understand if the transcriptional upregulation is attributable to H2BG53D occupancy, genome-wide distribution of FLAG-H2BG53D was mapped by CUT&RUN [34]. Gene set enrichment analysis revealed that upregulated genes (overlap between PRO-seq and RNA-seq) have higher FLAG enrichment in the FLAG-H2BG53D cell line, indicating the direct contribution of H2BG53D in transcriptional upregulation.

Among the list of upregulated genes with H2BG53D enrichment, *ANXA3* was chosen for validating the role of H2BG53D at the gene level. *ANXA3* has been implicated in the metastasis of liver cancer [35] and is overexpressed in a number of other cancers. Up-regulation of *ANXA3* at the transcriptional level was confirmed by determining the primary transcript level after DRB (5,6-Dichloro-1-beta-Ribo-furanosyl Benzimidazole) treatment. Furthermore, shRNA depletion of *ANXA3* dampens the migratory potential of H2BG53D cell lines. Most importantly, high *ANXA3* expression in PDAC patients is correlated with poor overall survival. Together, these findings support a model in which H2BG53D promotes the expression of cancer-associated genes as a result of weakened nucleosomal DNA histone interaction and, subsequently, increased transcription mediated by RNA polymerase II.

### 2.5. Other Histone-DNA Contact Point Mutations

The restriction enzyme accessibility assay performed by Biggert et al. [30] suggested that mutations occurring at histone-DNA contact points, including H2BG53D, converge functionally to enhance nucleosome sliding. Examples of these histone-DNA contact points include H2AR29 and H4R45 (Table 1).

H2AR29 is the second most frequently mutated residue on H2A, identified in 0.06% of patients on corporeal examination. H2AR29P and H2AR29Q accounted for 36% and 56% of all H2AR29 mutations, respectively, and were identified mainly in cervical and bladder cancer [24]. Since the methylation of H2AR29 has been implicated in transcriptional repression [36], it is reasonable to speculate that H2AR29 mutations might lead to aberrant gene activation through the disruption of histone methylation.

H4R45 mutations were identified in 0.02% of cancer patients [24]. H4R45 mutations were initially identified in yeast as a Sin mutation [37] (i.e., switch-independent mutations that alleviate the need for the SWI/SNF remodeling complex in regulating gene expression in yeast). The H4R45C mutant eliminates the residue’s interaction with nucleosomal DNA at the dyad axis [38] and has been shown to reduce RNA Pol II pausing on the nucleosome [39]. While H4R45C does not alter nucleosomal structure [38], it has been shown to affect Mg^2+^-dependent higher-order folding of the chromatin array [40]. The expression of H4R45C/H mutants has been associated with enhanced nucleotide excision repair and renders yeast cells more resistant to DNA damage [41].

## 3. H2BE76 Mutations

H2BE76 is the most frequently mutated H2B residue across all cancer types [23,24]. According to Bennett and colleagues’ analysis [24], polymorphism at E76 is 100 times more common in cancer patients compared to the general population (dbSNP) [42]. Moreover, E76 alterations co-occur with mutations of oncogenes such as *RAS, KDM6A, KMT2C*, and *TP53* at frequencies greater than chance events [24].

**Figure 5:**
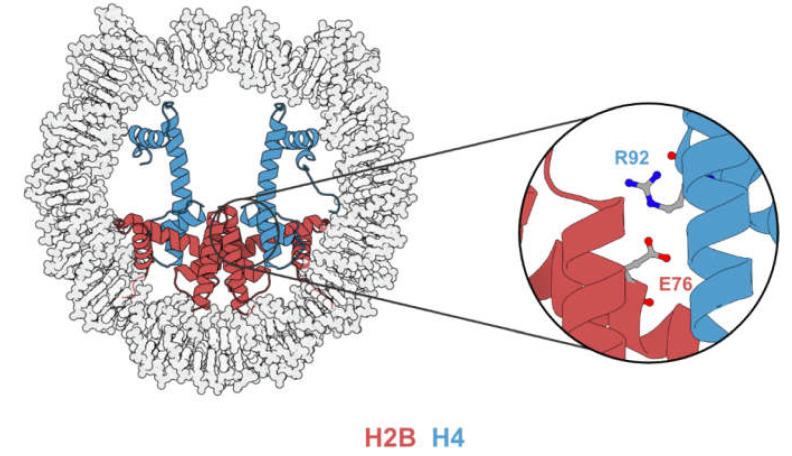
Illustration highlighting the H2B–H4 interface. H2BE76 locates in the inner region of the nucleosome and interacts with H4R92 (PDB code 2CV5).

The E76 residue is buried inside the nucleosome and is situated on the dimer-tetramer interface (Figure 5), suggesting that E76 mutants might contribute to tumorigenesis by nucleosome destabilization. Indeed, both in vitro analysis [23,24,43] and in vivo experiments [43,44] have demonstrated that mutations occurring at the E76 residue would destabilize the H2B–H4 interaction. Although the mechanistic link between nucleosome destabilization and alteration in gene expression remains elusive, the expression of E76 mutants in various cell lines leads to phenotypic change and gene expression profiles distinct from their wild-type counterparts.

### 3.1. H2BE76-to-K Mutation

H2BE76K accounts for 73.3% of all E76 alterations and was identified in 0.105% of cancer patients on cBioPortal. It was catalogued in a wide array of tumors including, but not limited to, bladder, breast cervical, lung, and ovarian cancer [24].

### 3.2. H2BE76K Disrupt H2B–H4 Interaction

H2BE76 forms a salt bridge with H4R92 [27] that is disrupted by H2BE76K. Arimura et al. [43] determined and superimposed the crystal structure of the E76K nucleosome with that of a canonical nucleosome. While the glutamate to lysine mutation does not affect the overall backbone geometries of H2A and H3, the side chain of the positively charged lysine causes electrostatic repulsion with H4R92, leading to a drastic conformational change in the α3-helix of H4. This was later corroborated by the molecular dynamic simulation performed by Bennett et al. [24], showing the disruption of the H2B–H4 association by H2BE76K. Since the H2B–H4 interaction is important for the assembly of histone octamer, the disruption of the H2BE76-H4R92 salt bridge is accompanied by nucleosome destabilization.

### 3.3. H2BE76K Nucleosomes Are Unstable

The instability of the H2BE76K nucleosome as a result of the disrupted H2B–H4 interaction is well supported by in vitro studies. In the absence of DNA, the H2A-H2BE76K dimer fails to be assembled onto the H3-H4 tetramer to form an octamer [24,43]. In the presence of DNA, the reconstitution of the nucleosome with H2BE76K is possible, but the resulting nucleosome releases the H2A-H2B dimer at a lower temperature [43] and is more sensitive to micrococcal nuclease (MNase) digestion compared to its wild-type counterpart [24]. Bagert et al. [30] later reported nucleosome instability in both heterotypic and homotypic H2BE76K nucleosomes, with the former being a likely representation of a physiological condition

As a result of the instability of the H2BE76K nucleosome, H2A-H2BE76K dimers were observed to be more mobile than their wild-type counterparts. Bagert et al. [30] conducted a high throughput Nap1-mediated histone exchange assay in order to investigate how histone core mutations affect histone exchange rate. In this assay, Nap1 (Nap1 is a histone chaperone responsible for both dimer and tetramer exchange in vivo; Bagert and colleagues have verified that no tetramer exchange occurred under their assay condition) and biotinylated dimers were incubated with a library of uniquely barcoded mono-nucleosomes. The mixture was then subjected to streptavidin purification in order to retrieve the mono-nucleosomes that have incorporated the biotinylated dimer. Finally, nucleosomal DNA was purified, and the barcoded reads were obtained by next-generation sequencing. More barcoded reads corresponding to the H2BE76K nucleosome were obtained compared to wild-type nucleosome, indicating a higher mobility histone exchange rate in the H2BE76K nucleosome.

The instability and elevated histone exchange of the H2BE76K nucleosome were further confirmed by in vivo experiments. Arimura et al. [43] conducted an immunoprecipitation experiment to examine the stoichiometry of the H2BE76K nucleosome in vivo. The immunoprecipitation was performed on HeLa cell lines expressing GFP-H2B or GFP-H2BE76K, with the nucleosome fraction treated with MNase. The successful immunoprecipitation of GFP-H2BE76K indicated that it was incorporated into the chromatin in vivo. Moreover, the amount of endogenous H2B pull-down by GFP-H2BE76K was reduced, indicative of nucleosome instability. Arimura and colleagues [42] furthered their investigation by comparing the histone exchange dynamics of GFP-H2B with those of GFP-H2BE76K by FRAP (fluorescence recovery after photobleaching). Half of a nucleus was first bleached by laser, and the subsequent recovery of the fluorescence signal as a result of GFP-H2B/GFP-H2BE76K diffusion into the bleached area was then recorded. HeLa cells expressing GFP-H2BE76K were reported to have fast recovery kinetics after photobleaching. This finding was later corroborated by a complementary FRAP assay. Bennett and colleagues [24] reported a higher recovery rate of GFP-H2A in MCF10A cells expressing either H2B or H2BE76K, supporting the notion that H2BE76K promotes mobility of H2A-H2B dimer.

### 3.4. H2BE76K Induces Transcriptional Alteration and Oncogenic Phenotypes

Growth defects accompanying H2BE79K expression in yeast [24] (H2BE79 in yeast is homologous to H2BE76 in humans) garnered evidence for the proposition that H2BE76K induces transcriptional alteration. Indeed, RNA-seq performed on both human [24,44] and mouse [30] cell lines showed that H2BE76K cells exhibit a distinct gene expression profile. Colony formation and soft agar assays conducted with various cell lines have uniformly demonstrated the enhanced colony formation ability of H2BE76K cells [24,43,44] (Table 2). Moreover, Bagert et al. [30] reported a differentiation blockade induced by H2BE76K expression in murine mesenchymal progenitor cells, the details of which will be discussed in Section 3.9.

The robustness of H2BE76K-induced transcriptional alterations across different cell lines is consistent with the mutation being identified in a wide range of tumor types, suggesting that H2BE76K might be acting through conserved cellular machineries. However, this does not exclude the possibility that H2BE76K might be cooperating with other oncogenes in a tumor type-specific manner.

### 3.5. H2BE76K Alters Chromatin Structure and Facilitates Transcription of Its Target Gene

There is ample evidence supporting the nucleosome destabilization and transcriptional alterations associated with H2BE76K. However, the mechanistic link between the two remains unclear. Current studies paint a picture where H2BE76K shapes the transcriptome by facilitating transcription of its target genes and altering chromatin accessibility.

Bennett and colleagues [24] observed increased sensitivity to MNase in both yeast and mammalian cells expressing H2BE76K, indicating that H2BE76K might alter chromatin accessibility. Chromatin accessibility profiling by ATAC-seq revealed a subset of open chromatin regions that are unique to H2BE76K cells. Approximately 40% of these regions were annotated to enhancers and 20% to transcriptionally inactive regions such as heterochromatin. Intriguingly, 3200 genes, which gained accessibility in their promoter regions, were reported to have higher expression (not to be confused with differentially expressed genes in RNA-seq) in H2BE76K-MCF10A compared to H2B-MCF10A, suggesting that the gain in chromatin accessibility might account for the transcriptional alteration in H2BE76K cells.

While Bennett and colleagues’ work has provided insight into how altered chromatin accessibility in H2BE76K cells is associated with gene expression, the direct effect of H2BE76K on transcription was unknown at the time. To understand the primary consequence of H2BE76K incorporation into the chromatin, Kang and colleagues [44] first profiled the genome-wide distribution H2BE76K to identify regions with H2BE76K enrichment.

CUT&RUN [34] profiling of FLAG-H2BE76K in MDA-MB-231 cells identified more than 2000 genes with significant H2BE76K enrichment. Next, to determine if there is any association between H2BE76K enrichment and transcriptional alteration, upregulated genes in H2BE76K-MDA-MB-231 cells were matched against a gene list ranked by H2BE76K enrichment in gene set enrichment analysis (GSEA). GSEA revealed overrepresentation of the upregulated genes among H2BE76K-enriched genes, which is indicative of the correlation between H2BE76K localization and transcriptional output.

Indeed, further examination of the transcription of ADAM19, an upregulated gene with H2BE76K enrichment, revealed elevated transcriptional activity. Together with the finding that H2BE76K promoted the mobility of the H2A-H2B dimer [21,28], this supports a model in which H2BE76K enhances transcription locally by facilitating the displacement of the H2A-H2B dimer during transcription.

### 3.6. Altered Interaction with Histone Chaperones and Specific Targeting of the Genic Region

In addition to demonstrating the link between H2BE76K enrichment and transcriptional upregulation in H2BE76K cell lines, profiling of H2BE76K distribution also unveiled specific targeting of the mutant histone into the chromatin. CUT&RUN revealed a distinct distribution of FLAG-H2BE76K compared to that of FLAG-H2B. More than 70% of the FLAG-H2BE767K peaks were annotated to a genic region, in stark contrast to the 30% observed for FLAG-H2B. The mechanism underlying the specific targeting of H2BE76K is currently unknown. Immunoprecipitation performed by Kang et al. [44] showed that the glutamate to lysine substitution enhances H2B interaction with NAP1L1 and NAP1L2. On the contrary, interaction with SPT16 was dampened. Although the biological relevance of these phenotypes has not been studied in depth, they suggested that investigation into the interactome of H2BE76K might uncover mechanism governing its deposition.

### 3.7. H2BE76-to-Q Mutation

H2BE76Q accounts for 21.7% of H2BE76 mutations and was found in 0.03% of all patient samples on cBioPortal. Cancers with H2BE76Q mutation include, but are not limited to, lung, breast, cervical, and uterine cancers [24]. Both H2BE76K and H2BE76Q give rise to unstable nucleosomes and lead to similar growth defects when expressed in yeast [24]. However, recent transcriptomic profiling has revealed that the gene expression profile of H2BE76Q-expressing cells is distinct from that of H2BE76K-expressing cells [30].

### 3.8. H2BE76Q Nucleosomes Are Unstable and Lead to Gene Dysregulation in Yeast

In vitro studies showed that the stability of the H2BE76Q nucleosome is an intermediate between that of H2BE76K and wild-type nucleosomes. Unlike H2BE76K which fails to form an octamer altogether in the absence of DNA, H2BE76Q is able to form an octamer albeit with lower efficiency compared to wild-type H2B [24]. As reported by Bennett et al. [24] and Bagert et al. [30], the expression of H2BE76Q or H2BE79Q in yeast leads to growth defects.

### 3.9. H2BE76K and H2BE76Q Expression in Mesenchymal Progenitors Resulted in Different Cell Fates

H2BE76Q expression in mesenchymal progenitor cells results in transcriptomic alterations distinct from those caused by H2BE76K [30]. Bagert and colleagues [30] performed RNA-seq on mesenchymal progenitor cells stably expressing H2BE76Q and identified more than 2000 differentially expressed genes, 40% of which overlapped with those from H2BE76K-expressing mesenchymal progenitor cells. KEGG pathways unique to H2BE76Q cells include “GAG biosynthesis”, “proteoglycans in cancer”, and “protein digestion”.

Bagert and colleagues [30] speculated that H2BE76 mutants expression might alter cell fate, since the genes regulating stem cells pluripotency are upregulated in both H2BE76K and H2BE76Q cells. To examine the effect of H2BE76 mutants’ expression on cell differentiation, C3H10T1/2 expressing H2BE76K/Q (the same cell lines used for RNA-seq) were subjected to an adipocyte and chondrocyte differentiation assay. C3H10T1/2 is a robust and established model system used to study cell differentiation, the same system that demonstrated differentiation blockade induced by H3K36M [15]. Interestingly, while H2BE76K expression inhibits differentiation into adipocytes and chondrocytes, H2BE76Q expression shows little to no effect.

While both H2BE76K and H2BE76Q lead to nucleosome instability, Bagert and colleagues’ [30] work on H2BE76 mutations suggests that there is an additional mechanism governing the transcriptional alterations associated with H2BE76 mutations. The aforementioned immunoprecipitation performed by Kang et al. [44] demonstrated that H2BE76K and H2BE76Q affect interactions with different histone chaperones, indicating that the two mutations likely have distinct interactomes which could explain the gene expression changes unique to both mutations.

### 3.10. Other Nucleosome-Destabilizing Histone Mutations

H2BE76 mutants represent a class of nucleosome-destabilizing histone mutants. In vitro studies conducted by Bagert et al. [30] have uncovered additional nucleosome-destabilizing mutations which converge on the dimer-tetramer interface, including H2BD68N, H2BE71K/Q, and H4K91N/R.

H2BD68 mutations occurred in 0.05% of cancer patients on cBioPortal, 52% of which result in a D to N substitution identified mainly in lung and uterine cancer [24]. Like H2BE76, H2BD68 also interacts with H4R92 [23]. The expression of H2BD68A/N (or its yeast counterpart H2BD71A) has been shown to prevent growth in yeast [30,45]. In addition, H2BD68A has been shown to impair the binding of H2A.Z-H2B dimers to the chromatin [46].

H2BE71 mutations occurred in 0.06% of cancer patients on cBioPortal, 96% of which led to an E-to-K or E-to-Q substitution. H2BE71K was identified mainly in breast and skin cancer, whereas H2BE71Q was predominantly found in lung cancer [24]. According to Bagert et al. [30], H2BE71K/Q is similar to H2BE76K/Q in two aspects. Transcriptionally, the gene expression profile of H2BE71K-expressing mesenchymal progenitor cells is distinct from that of H2BE71Q-expressing mesenchymal progenitor cells. Phenotypically, only H2BE71K impairs differentiation of mesenchymal progenitor cells into adipocytes.

H4K91 mutations occurred in 0.009% of cancer patients on cBioPortal [24]. H4K91 interacts with H2BE71 to form a salt bridge at the dimer-tetramer interface [23]. Acetylation, mono-ubiquitination, and glutarylation on H4K91 have been shown to regulate chromatin structures and DNA damage response [47,48,49]. Apart from cancer, H4K91 mutations have also been implicated in severe developmental diseases by altering cell cycle control and responses to DNA damage [50,51].

## 4. Acidic Patch Mutations

**Figure 6:**
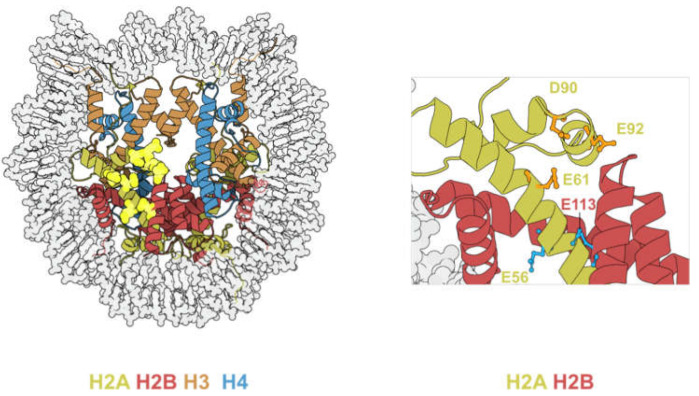
Left: Illustration showing the location of the acidic patch. The eight acidic patch residues are represented by bright yellow spheres. Right: Detailed view of acidic patch residues whose mutations have been shown to affect nucleosome sliding. Residues that inhibit nucleosome sliding when mutated are highlighted in orange. Residues that promote nucleosome sliding when mutated are highlighted in blue (PDB code: 2CV5).

The acidic patch refers to a group of glutamate and aspartate residues on H2A and H2B that form a highly negatively charged groove on the surface of the nucleosome [1] (Figure 6, left panel).

A common structural motif is found on all crystal structures of nucleosome-bound chromatin factors. This shared motif is called an “arginine-anchor”, defined as a single arginine residue that inserts into a pocket generated by H2AE61, D90, and E92 [52]. Owing to such versatility, the acidic patch can accommodate a wide range of motifs such as α-helices, loops, and hairpins [53]. Hence, acidic patch-binding proteins are involved in a plethora of biological activities (summarized in Table 3).

The acidic patch consists of six H2A residues (E56, E61, E64, D90, E91, E92) and two H2B residues (E105, E113), all of which are frequently mutated in cancer (Table 4) [23]. Among the eight residues, H2BE113 mutations occur at the highest frequency, and is the second most mutated H2B residue after H2BE76 [23].

### 4.1. H2B E113 Mutations Promote Chromatin Remodeling and Alter Transcription

H2BE113 mutations occurred in 0.07% of all of the cancer patients on cBioPortal. H2BE113K/Q accounts for 93.3% of all H2BE113 mutations and was mainly identified in breast and bladder cancer [24].

H2BE113 has been shown to interact with subunits of ATP-dependent chromatin remodeling complexes such as SMARCB1 [58] and SMARCA5 [59]. Both H2BE113K and H2BE113Q have been shown to promote SMARCA5-mediated nucleosome remodeling based on in vitro studies [28,50], hinting at a possible alteration to the chromatin structure and thus gene expression (refer to Section 4.2 for further discussion). While the pathogenicity of H2BE113 mutants is yet to be studied in cancer cells, the expression of H2BE113K/Q in mesenchymal progenitor cells results in a change in gene expression. Pathway enrichment analysis of the upregulated genes revealed enrichment in “pathways in cancer”, “focal adhesion”, and the “Rap1 signaling pathway”. Similar to H2BE76 mutations, only the E-to-K mutant impairs differentiation into adipocytes/chondrocytes. This observation could be an indication that charge swapping mutants are more disruptive to acidic patch function, leading to greater extent of gene mis-regulation and subsequent oncogenic cell fate.

### 4.2. Acidic Patch Mutations Affect Nucleosome Sliding

**Figure 7:**
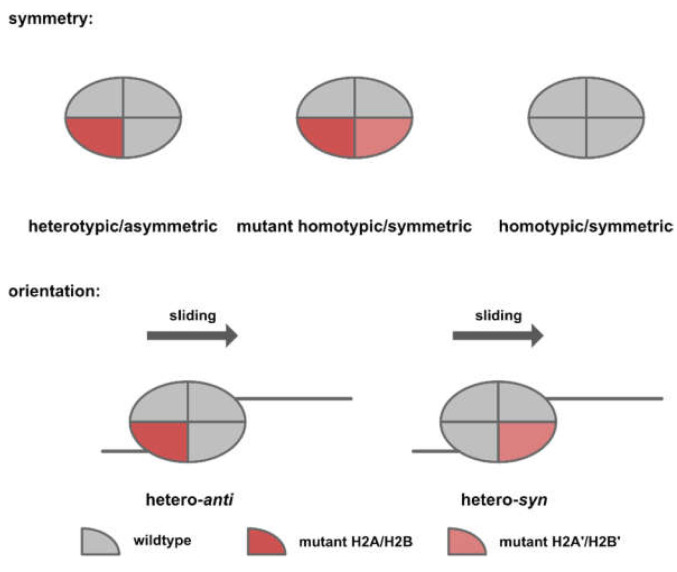
Cartoon illustration of nucleosome symmetry and orientations.

Based on the in vitro experiments conducted by Dao et al. [59] and Bagert et al. [30], acidic patch mutations converge functionally to affect nucleosome sliding. Dao and colleagues [59] conducted an SMARCA5-dependent restriction enzyme accessibility assay and reported an inhibitory effect for mutations occurring within the arginine pocket (H2AE92K, H2AD90N, and H2AE61D). On the contrary, mutations distal to the arginine pocket (H2AE56Q, H2AE56K, and H2BE113K) were reported to have a stimulatory effect (Figure 6, right panel). This finding was later reproduced by Bagert and colleagues [30] in a high-throughput SMARCA5-dependent restriction enzyme accessibility assay. Notably, the direction and kinetics of the chromatin remodeling event was determined by both the symmetry and orientation of the target nucleosomes. Owing to the polygenic nature of human histones, expression of a mutant histone from one allele would likely result in a mixture of symmetric/homotypic (wild-type-wild-type or mutant-mutant) and asymmetric/heterotypic (wild-type/mutant) nucleosomes in cancer cells. Since nucleosome sliding is a directional process, asymmetric nucleosomes could assume two orientations, depending on the relative position of the chromatin remodeler (Figure 7).

**Figure 8:**
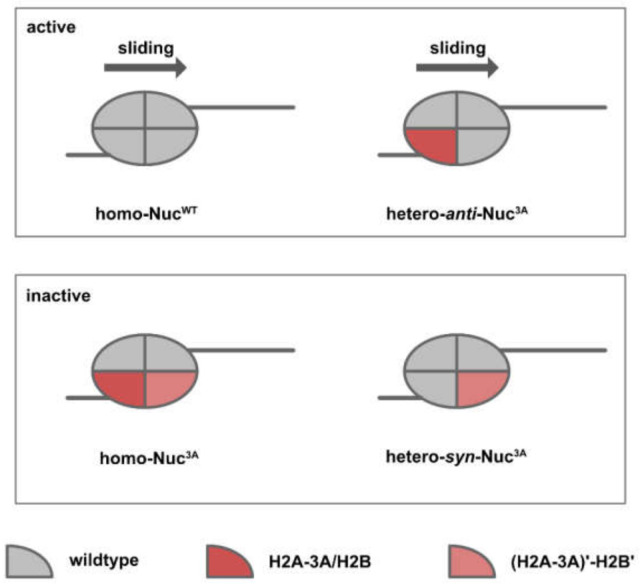
Cartoon illustrating the result of chromatin remodeling assay conducted by Dao et al. [59]. Heterotypic nucleosomes of opposite orientations behave differently in SMARCA5-dependent chromatin remodeling assay. Hetero-*anti*-Nuc^3A^ (mutant histone on the opposite side of DNA overhang) shows similar sliding as wildtype nucleosome, while hetero-*syn*-Nuc^3A^ and homo- Nuc^3A^ are completely inactive. H2A-3A, triple H2A mutant of E61A, D90A, E92A.

Dao and colleagues [59] reconstituted asymmetric nucleosomes of a specific orientation in order to examine how nucleosome asymmetry might affect the kinetics and direction of chromatin remodeling (Figure 8). For acidic patch mutations that inhibit chromatin remodeling (Nuc^3A^, triple mutant for H2A E61A, D90A, E92A), heterotypic nucleosomes of opposite orientations resulted in distinct remodeling kinetics. While homo-Nuc^3A^ and hetero-*syn*-Nuc^3A^ were completely inactive, hetero-*anti*-Nuc^3A^ and homo-Nuc^WT^ exhibited similar sliding kinetics. For H2BE113K, which has been shown to promote nucleosome sliding, the stimulatory effect was only observed in hetero-*syn*-Nuc^H2BE113K^.

In vitro studies from Dao et al. [59] and Bagert et al. [30] have characterized the effect of acidic patch mutations on SMARCA5-dependent nucleosome sliding. The validation of these phenotypes in a cell-based system is foreseeably challenging because the symmetry and orientation of the nucleosome would have to be considered in future studies.

## 5. Conclusions

Previously, oncohistones referred exclusively to H3 mutants, namely H3K27M, H3G34V/R, and H3K36M. These classical oncohistones are restricted to several types of cancers and promote tumorigenesis mainly through the perturbation of gene transcription via alterations in histone modifications. Recent reports have demonstrated that histone mutations are more prevalent and affect a wider range of cancers than previously appreciated. Contrasting to classical oncohistones, which affect post-translationally modified tail residues of histones, many of the newly identified mutations occur on the residues in the histone fold domains and are not known to be modifiable.

**Figure 9:**
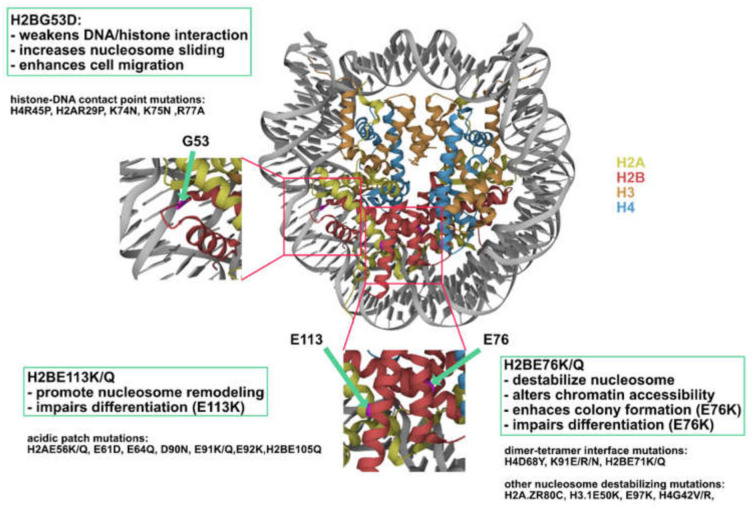
H2B mutations represent three novel classes of oncohistones. Nucleosome (PDB code 2CV5) showing the location of H2BG53, E76, and E113 (highlighted in magenta).

Although many of the newly identified histone mutations may ultimately be passenger [23], recent studies have defined new classes of oncohistones (Figure 9): (1) Histone-DNA contact point mutations that promote nucleosome sliding, represented by H2BG53D, which have been shown to promote the transcription of migration-related genes; (2) Nucleosome-destabilizing mutations, the majority of which converge onto the dimer-tetramer interface (H2BE76K belongs to this class of mutations and has be shown to alter chromatin accessibility in vivo); and (3) Acidic patch mutations that affect chromatin remodeling and are represented by H2BE113K, the expression of which resulted in differentiation impairment in mesenchymal progenitor cells.

Among the three mutations (H2BG53D, H2BE76K, and H2BE113K), the role of H2BG53D and H2BE76K on chromatin functions and gene expression are better characterized. For H2BE113K, while its effect on nucleosome sliding has been well-documented, further investigation is required to establish its mechanistic link with changes in gene expression.

## Figures and Tables

**Table 1:** Histone-DNA contact point mutations.

Residue	Cancer	Post-Translational Modification	Frequencies in Cancer	Oncogenic Mechanism (Proposed)
H2BG53	Pancreas, brain, prostate, lung	No	0.0007	Weakens interaction between nucleosomal DNA and increased RNAPII passaging [31], upregulation of cancer and migration-associated genes [32]
H2AR29	Cervix, bladder	Methylation, transcriptional repression [36]	0.0006	(Aberrant activation of gene expression)
H4R45	Esophagus, uterus, colon, lung	No	0.0002	(Alteration in higher-order chromatin structure, resistance to DNA damages)

**Table 2:** Cell-based assay and RNA-seq conducted on H2BE76K cell lines.

Cell Type	Assay Condition	RNA-seq	Reference	Remark
NIH3T3; Mouse embryonic fibroblast	Transient expression; colony formation assay	No	[43] Arimura et al.	
MCF10A; Non-tumorigenic mammary epithelial cells	Stable expression; soft agar assay	Yes	[24] Bennett et al.	H2BE76K enhances colony formation in cooperation with oncogenic PIK3CA-H1047R
MDA-MB-231; Breast cancer cells	CRISPR/Cas9 knockin; colony formation assay	Yes	[44] Kang et al.	
C3H10T1/2; Mouse mesenchymal progenitors	Adipocyte and chondrocyte differentiation assay	Yes	[30] Bagert et al.	Experiments were conducted for both H2BE76K and H2BE76Q cell lines

**Table 3:** Table of the functions of acidic patch-binding protein, listing some examples of acidic patch-binding proteins and their known interacting residues on the acidic patch. * Residue number following human histones.

	Function	Interacting Residue *	Reference
53BP1	DNA damage repair; reader of H2AK15ub	H2AE61, E91, E92 H2BE105	[54]
DOT1L	Histone methyltransferase of H3K79me3; H3K79me3 is correlated with active transcription	H2AE61, E64, H2BE113	[55]
RNF168	DNA double strand break repair; ubiquitin ligase for H2AK13, K15	H2AE64, E91, E92, H2BE105	[56]
HMGN2	Chromatin decompaction to enhance transcription	H2AE61, E64, D90, E91, E92, H2BE105	[57]
SMARCB1	Chromatin remodeling	H2AE64, E91, H2BE113	[58]

**Table 4:** Frequency of acidic patch residues in cancer.

	Residue	Frequency in Cancer (×10^−4^) (Bennett et al. [24])
H2A	E56	3.59
	E61	1.92
	E64	1.2
	D90	3.11
	E91	2.88
	E92	2.64
H2B	E105	2.64
	E113	7.19

## Abbreviations

**Abbreviation (In Order of Appearances)**	**Definition/Remarks**
H3K27M	H3 Lysine 27 to Methionine
H3G34V/R	H3 Glycine 34 to Valine/Arginine
H3K36M	H3 Lysine 36 to Methionine
H3K27me3	H3 Lysine 27 tri-methylation; repressive histone mark
H3K36me3	H3 Lysine 36 tri-methylation; active histone mark
EZH2	Enhancer of Zeste 2; subunit of PRC2 complex, histone lysine methyltransferase for H3K27me3
SETD2	SET Domain Containing 2; histone lysine methyltransferase for H3K36me3
